# Reliability and Validation Study of the Spanish Translation of the Nociception Coma Scale-Revised—Adapted for Intubated Patients (NCS-R-I)

**DOI:** 10.3390/nursrep15080278

**Published:** 2025-07-30

**Authors:** Candelas López-López, Gemma Robleda-Font, María del Mar Sánchez-Sánchez, Carmen María Sarabia-Cobo, Ignacio Latorre-Marco, Montserrat Solís-Muñoz, Teresa Pérez-Pérez, Cristina Martín-Arriscado Arroba, Caroline Schnakers, Juan Roldan-Merino

**Affiliations:** 1 Hospital Universitario 12 de Octubre, 28041 Madrid, Madrid, Spain; 2 Instituto de Investigación Sanitaria Hospital 12 de Octubre (imas12), 28041 Madrid, Madrid, Spain; 3 Faculty of Nursing, Physiotherapy and Podiatry, Complutense University of Madrid, 28040 Madrid, Madrid, Spain; 4 Faculty of Medicine and Health Sciences, Universitat Internacional de Catalunya, 08195 Sant Cugat del Vallés, Barcelona, Spain; 5 Centro Cochrane Iberoamericano, Hospital de la Santa Creu i Sant Pau, 08025 Barcelona, Barcelona, Spain; 6 Hospital Universitario de Getafe, 28905 Getafe, Madrid, Spain; 7 Faculty of Nursing, Universidad de Cantabria, 39008 Santander, Cantabria, Spain; 8 Hospital Universitario Puerta de Hierro Majadahonda, 28222 Majadahonda, Madrid, Spain; 9 Instituto de Investigación Sanitaria Hospital Puerta de Hierro-Segovia Arana, 28222 Majadahonda, Madrid, Spain; 10 Faculty of Statistical Studies, Complutense University of Madrid, 28040 Madrid, Madrid, Spain; 11 Hospital Research Institute, Casa Colina Hospital and Centers for Healthcare, Pomona, CA 91767, USA; 12 Faculty of Nursing, Campus Docent Sant Joan de Déu, 08830 Sant Boi de Llobregat, Barcelona, Spain

**Keywords:** nursing, intensive care units, brain injuries, pain, behavior

## Abstract

**Background/Objectives**: Pain assessment scales provide a clear clinical benefit in patients who are unable to self-report. The Nociception Coma Scale-Revised—adapted for Intubated patients (NCS-R-I) was developed to assess pain in patients with acquired brain injury who are unable to self-report. However, this instrument has not yet been translated and validated for use in Spain. The objective was to translate the Nociception Coma Scale-Revised—adapted for Intubated patients (NCS-R-I) into Spanish and to assess the reliability and validity of the Spanish version in patients with brain injury. **Methods**: This study was carried out in two phases. First, the scale was translated into Spanish. Next, a psychometric analysis was performed to determine the reliability and validity of the Spanish version of the NCS-R-I in 207 critically ill patients with acquired brain injury and disorders of consciousness. Two blinded observers administered the scale at three time points: 5 min before, during, and 15 min after a series of nociceptive and non-nociceptive procedures. **Results**: The internal consistency of the NCS-R-I was acceptable (ordinal alpha = 0.60–0.90). Interobserver agreement was good (kappa = 0.80; intraclass correlation coefficient = 0.90). In terms of discriminant validity, the AUC was 0.952 (95% CI: 0.931–0.973). NCS-R-I scores increased significantly during performance of nociceptive procedures compared to scores obtained before and after these procedures, confirming the scale’s sensitivity to change. Similarly, during the performance of nociceptive procedures, scores on the NCS-R-I were significantly higher (*p* < 0.001) than those observed during non-nociceptive procedures. **Conclusions**: The results of this study demonstrate that the NCS-R-I is a valid, reliable tool for the assessment of pain in patients with acquired brain injury who are unable to self-report.

## 1. Introduction

Pain is common in critically ill patients admitted to the intensive care unit (ICU) [1,2] and is mainly attributable to the injury or illness, or to treatment-related procedures [3,4,5]. Uncontrolled pain can lead to agitation, delirium, and stress, which can increase the risk of chronic pain [6,7,8,9]. Consequently, an accurate assessment of the patient’s level of pain is crucial to select the most appropriate pain management measures. While self-report measures are considered the gold standard for pain assessment, many ICU patients are unable to self-report. In these cases, behavioral pain scales are recommended to assess pain levels [10,11].

Critically ill patients with acquired brain injury are characterized by the presence of disorders of consciousness. In many cases, these patients require placement of an artificial airway and sedation. These clinical features compromise the patient’s capacity to reliably self-report their pain experience. Moreover, pain behavior may be underestimated due to alterations caused by the injury (e.g., focal neurological signs), and the specific pain behaviors exhibited by these patients [12,13,14]. In this population, untreated pain, in addition to the aforementioned consequences, can lead to increased intracranial pressure, decreased venous flow, and greater cerebral metabolism [15].

Until relatively recently, no behavioral pain scales were available to assess pain in patients with acquired brain injury. In fact, the Nociception Coma Scale (NCS)—developed by Schnakers et al. in 2010 [16]—was the first pain scale specifically developed to evaluate nociceptive behavior in patients with acquired brain injury. The original NCS contains four domains (motor response, verbal response, facial expression, and visual response) with four items per domain. The NCS validation study found significant differences in total scores depending on the pain threshold (non-painful, mild, moderate) for the procedure (Cohen’s kappa = 0.61). In 2012, Chatelle et al. [17] published a revised version of the scale, the NCS–Revised (NCS-R), which omitted the visual response domain, thereby increasing the sensitivity of the scale from 46% to 83–96%. More recently, in 2019, Bernard et al. [18] adapted the NCS and NCR-R for use in intubated patients. The new scale, called the Nociception Coma Scale-Revised—adapted for Intubated patients (NCS-R-I), omits the verbal response domain. The psychometric analysis of the NCS-R-I showed a Cronbach’s alpha of 0.69 (0.64–0.73), a kappa of 0.84 (0.81–0.87), and an area under the curve (AUC) (discriminant validation) of 0.97 (0.95–0.99). Those findings confirmed the validity and reliability of this scale to measure nociceptive behavior in patients with acquired brain injury and an artificial airway.

Given the potentially major negative implications of failing to adequately detect and treat pain in ICU patients with acquired brain injury and disorders of consciousness, there is a clear and urgent need to develop scales designed specifically to evaluate pain in this patient population. Currently, there is a notable lack of specific pain assessment tools for use in this highly vulnerable population [19,20,21]. In this regard, the NCS-R-I scale is a relatively new and promising tool for pain assessment in this population. However, the NCS-R-I has only been available since 2019 and has not yet been translated or adapted for implementation in routine clinical practice in Spain.

In this context, the objective of this study was to translate the NCS-R-I into Spanish and to evaluate the reliability and validity of the Spanish language version in patients with acquired brain injury and an artificial airway who are unable to self-report pain.

## 2. Materials and Methods

### 2.1. Study Design

The study was carried out in two phases. First, the NCS-R-I scale was translated into Spanish. Next, a psychometric analysis was performed to validate the NCS-R-I in accordance with the recommendations of COnsensus-based Standards for the selection of health Measurement INstruments (COSMIN), Amsterdam, The Netherlands [22].

#### 2.1.1. Phase 1: Spanish Translation of the NCS-R-I and Pilot Test

Permission was previously obtained from C. Schnakers, the first author of the original scale (NCS) [16], and from G. Chanques, the corresponding author of the NCS-R-I [18].

The translation/back-translation of the NCS-R-I into Spanish was carried out in four steps. First, two bilingual nurses whose native language was Spanish independently translated the scale from English to Spanish. Second, a group of experts compared and synthesized the two translations to create a single version. Third, two bilingual nurses whose native language was English performed the back translation. These nurses performed the translation independently, thus creating two versions, which were compared with each other and with the original version. Fourth, the expert group created the final Spanish-language version of the instrument after evaluating the semantic, idiomatic, and conceptual equivalence of the original English-language scale and the new Spanish-language version. The group of experts was comprised of five professionals with more than ten years of clinical experience in the care of critically ill patients with acquired brain injury and disorders of consciousness. Two of the experts had previous methodological experience in psychometrics.

After completing the Spanish version of the NCS-R-I, a pilot was conducted in a small sample (n = 20) before starting the study in order to thoroughly train all of the professionals involved in the project in the proper use of the NCS-R-I. The patients of the pilot were not a part of the sample described in the section below.

#### 2.1.2. Phase 2: Analysis of the Psychometric Properties of the Spanish Version of the NCS-R-I

A psychometric analysis was performed to assess the validity and reliability of the NCS-R-I in a sample of 207 critically ill patients with acquired brain injury (traumatic and non-traumatic) and disorders of consciousness.

### 2.2. Patients and Setting

The study was conducted in the ICUs of four university hospitals in Spain.

Inclusion criteria were as follows: age ≥ 16 years; acquired brain injury, inability to self-report by verbal or motor response; and presence of an artificial airway. Informed consent was obtained from the patient’s family member or representative. Exclusion criteria were: previous cognitive injury/impairment; any condition that limited or suppressed behavioral response such as severe polyneuropathy (diagnosed or suspected), brain death, muscle relaxants, barbiturate coma, deep sedation level (−5 on the Richmond Agitation Sedation Scale [RASS]), and a low level of consciousness (i.e., coma or 3 points on the Glasgow Coma Scale [GCS]).

### 2.3. Sample

The aim was to assess the discrimination capacity of the NCS-R-I scale to correctly classify patients with pain (during the nociceptive procedure) and patients without pain (non-nociceptive procedure). We estimated that a sample of 200 patients would provide a 95% confidence interval width of 0.04 for the AUC of the scale based on the expected good discrimination capacity, AUC = 0.95 [23].

### 2.4. Independent and Outcome Variables

The main outcome variable was the pain score on the NCS-R-I scale [18]. This scale consists of three domains: (1) facial expression, (2) motor response, and (3) compliance with ventilation. Each of the three domains includes four items, scored from 0 to 3, as follows: 0 points (i.e., no nociceptive behavioral response), and 1, 2, or 3 points depending on the observed nociceptive behavioral response. The total score on the NCS-R-I ranges from 0 to 9 points. Higher scores reflect greater intensity of the nociceptive behavioral response.

Demographic and clinical variables were obtained from the patients’ medical records and included the following: age and sex; etiological diagnosis of acquired brain injury; neurosurgery; focal neurological signs; Simplified Acute Physiology Score II [24]; days of invasive mechanical ventilation; number of days in the ICU; days until assessment; ICU mortality; and type and dose of analgesia and sedation administered continuously and as an intravenous bolus (eight hours prior to performing the study procedures).

Before performing the procedures, the RASS [25] was applied to assess the level of sedation. The RASS is a 10-point scale that measures five levels of sedation (−5 to −1) and four levels of agitation (+1 to +4). A score of 0 indicates the patient is calm and alert. The GCS was used to determine the level of consciousness [26]. The GCS evaluates three parameters: eye opening (4 response options), verbal response (5 options), and motor response (6 options). Total scores range from 3 to 15 points.

### 2.5. Procedure

Two observers independently applied the NCS-R-I to evaluate nociceptive response at three time points: 5 min before, during, and 15 min after the application of nociceptive and non-nociceptive procedures.

Two nociceptive procedures were performed: tracheal suctioning [3] and the application of pressure to the nail bed of the ring finger. If the latter procedure was not possible, pressure was applied to the nail bed of the middle finger on both hands [17]. The Algiscan plus^®^, Hagen, Germany, algometer was used to apply pressure (5 to 8 kg) until a behavioral response was obtained or for a maximum of 30 s. The non-nociceptive procedure (control procedure) consisted of using a gauze pad to rub an area of healthy skin tissue on the patient’s forearm (or calf if the forearm was not available) [27]. All three procedures were performed in all patients and a 15-min “washout period” was guaranteed between each procedure.

We registered the analgesia/sedation treatment used, the level of sedation, and the patient’s level of consciousness on the day of the evaluation.

### 2.6. Statistical Analysis

A descriptive analysis of the patients’ demographic and clinical characteristics and the NCS-R-I was carried out.

To assess the reliability of the NCS-R-I at the three study time points and for each procedure, internal consistency was calculated using the ordinal coefficient alpha [28], which is indicated for ordinal items and is based on the polychoric correlation matrix. The kappa coefficient for each item on the NCS-R-I was used to evaluate interobserver agreement. The intraclass correlation coefficient (ICC) with its 95% CI was obtained for the total score.

The discriminant validity, sensitivity to change, and convergent validity18 were assessed. The scale’s capacity to discriminate between nociceptive and non-nociceptive procedures was examined using the area under the ROC curve (AUC). The Friedman test was used to assess sensitivity to change between the three assessment points (before, during, and after each procedure). When significant differences were observed, 2 × 2 comparisons were performed using the Wilcoxon signed-rank test, corrected for multiplicity. Spearman’s correlation coefficient was used to assess convergent validity between the NCS-R-I and GCS.

The IBM-SPSS Statistics for Windows program (v. 28.0) [29] and the Factor software (v. 10.4) [30] were used for data analysis.

## 3. Results

### 3.1. Phase 1

The translation and back translation were performed successfully without any notable difficulties. The pilot study showed that the scale was easy to administer to evaluate procedure-related nociceptive response. Table 1 presents the original NCS-R-I scale and its semantic equivalence with the Spanish version of the NCS-R-I.

### 3.2. Phase 2

#### 3.2.1. Patient Characteristics

Table 2 shows the patients’ demographic and clinical characteristics. Most of the patients (66.7%) were men, with a median age of 60 years. The main aetiology of brain injury was stroke and traumatic brain injury. A total of 81 patients (39.1%) underwent neurosurgery. All patients received analgesia and/or intravenous sedation, delivered in continuous infusion or bolus, corresponding to a mild to moderate level of sedation (RASS-2). In the patients who received continuous infusion, the most common analgesic drugs were opioids (65.2%). On the day of the assessment, all patients had a low level of consciousness (GCS ≤ 9), and 52% (n = 106) presented focal neurological signs.

#### 3.2.2. Reliability

Table 3 shows the ordinal alpha values, the degree of interobserver agreement for each domain, and the ICC for the complete scale. As that table shows, the alpha values (internal consistency) ranged from approximately 0.65 to 0.90.

Interobserver agreement for each evaluation time point and procedure was good (kappa coefficient > 0.80 for all domains). The ICC for the total score was excellent (>0.90).

#### 3.2.3. Discriminant Validity and Sensitivity to Change

The NCS-R-I scores obtained by the two observers are shown in Table 4, showing scores according to the time point (before, during, and after the procedures) and type of procedure (nociceptive vs. non-nociceptive). Behavioral response pre- and post-procedure was low, with a median total score of 0 on the NCS-R-I for both time points. Similar results were observed for the non-nociceptive procedure. Behavioral response increased during the nociceptive procedures, with a median score of 5 during tracheal suctioning and 3 during left and right nail bed pressure. The highest scores were observed on domains 1 (motor response) and 3 (facial expression).

**Figure 1:**
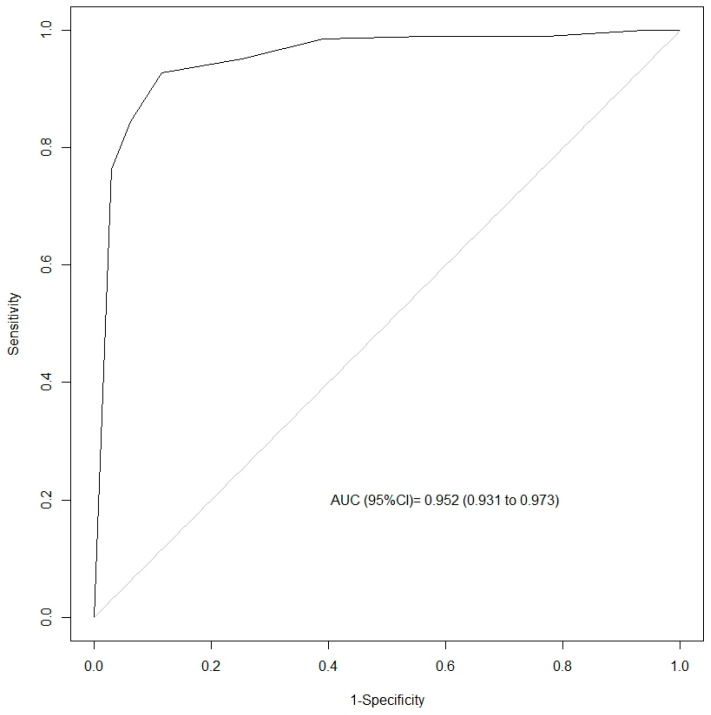
ROC curve of the NCS-R-I associated with pain during non-nociceptive versus nociceptive procedures. Abbreviations: AUC: Area Under Curve.

Figure 1 shows the ROC curve for the NCS-R-I scale. The AUC was 0.952 (95% CI: 0.931–0.973) during the nociceptive versus the non-nociceptive procedures.

As Table 5 shows, the NCS-R-I score increased significantly during the nociceptive procedures when compared to the pre- and post-procedure time points (Wilcoxon signed-rank test, *p* < 0.001).

#### 3.2.4. Convergent Validity

The NCR-R-I scores, obtained during the nociceptive procedures, showed significant but weak correlations with the GCS scores (*p* < 0.01; Spearman’s correlation coefficient was 0.5 for tracheal suctioning and 0.2 for nail bed pressures).

## 4. Discussion

The objective of this study was to translate the NCS-R-I—a scale designed to evaluate nociceptive behavior in patients with acquired brain injury—into Spanish and to evaluate the psychometric properties of the Spanish version. To our knowledge, this is the first time that a psychometric analysis of this scale has been performed since the initial publication by Bernard et al. [18]. The current psychometric study included a large sample (n = 207) of critically ill patients with acquired brain injury and disorders of consciousness admitted to four hospital ICUs in Spain. Our findings show that the psychometric properties of the Spanish version of the NCS-R-I are comparable to those observed in the NCS-R-I validation study by Bernard et al. [18].

In terms of reliability, the internal consistency was adequate, with alpha values for most procedures above > 0.7 [30]. With regards to interobserver agreement, kappa index values were close to +1, indicating a nearly perfect correlation between observers (>0.80) [31,32]. The ICC was also excellent (>0.90) [32]. The reliability of the NCS-R-I was similar to that obtained in the original and revised NCS, both of which were designed for patients able to breathe independently (i.e., not on mechanical ventilation and without an artificial airway) [33,34,35]. Although the raters were given clear, standardized instructions for how and when to apply the scale (e.g., 5 min before, during, and 15 min after the procedures), the internal consistency was less than optimal. Although the reason for this is unclear, it may be related to the limited number of domains on the scale and/or to the response pattern in this patient population, in which only some of the behavioral responses (e.g., facial expression of pain) were observed during the procedures. The presence of focal neurological signs—present in 52% (n = 106) of the sample—may also have influenced behavioral expression. These results suggest that some of the domains may require slight modifications [36,37].

In terms of validity, the ROC curve (AUC) values (>0.90) were comparable to those reported by Bernard. et al. [18]. Criterion validity could not be assessed due to the lack of a gold standard. Similarly, we were unable to identify an adequate method to measure convergent validity. The correlation between the NCS-R-I and the GCS was poor, perhaps due to the low level of consciousness in this sample (GCS ≤ 9 in all patients). It is likely that a sample of brain injury patients with a moderate level of consciousness (GCS 9–12) would have led to more robust convergent validity values. In addition, if validated tools were available to assess a similar construct (nociception), this would have provided the most appropriate methodological framework. However, this finding is consistent with the study by Bernard et al. [18], who also found a poor correlation between the NCS-R-I and pupillometry, which may be because pupillometry has not been validated as a tool to assess nociception in patients with brain damage, as these patients may have an altered pupillary response.

With respect to sensitivity to change (a measure of validity), the NCS-R-I score increased significantly during the nociceptive procedures compared to the scores measured before and after these procedures. Similarly, NCS-R-I scores were higher during nociceptive procedures than during non-nociceptive procedures, in line with findings of other studies that have evaluated different versions of the scale [16,17,18].

The scores obtained during tracheal suctioning were comparable to those reported by Bernard. et al. [18], who reported scores of around 4–5 points. However, in the nail bed pressure procedure, scores on the NCS-R-I (patients on mechanical ventilation) ranged from 2 to 3 points in our study versus 4 to 5 points on the NCS-R (patients not on mechanical ventilation) in the study by Chatelle et al. [17]. These values may be due to the level of sedation of patients on mechanical ventilation, which in our study was mild to moderate, as this could lower behavioral response. The behavioral response depends on the specific nociceptive procedure. For example, the domain “compliance with ventilation” was observed less frequently during the application of pressure to the right and left nail beds than during tracheal suctioning. In the NCS-R, other authors have reported a similar pattern for the equivalent domain (verbal response), which is the domain least activated during nociceptive stimulation (mainly nail bed pressure) [17,23,38,39].

Previously, Chatelle et al. [40] described their experience with the NCS-R in non-mechanically ventilated patients with brain injury. In that study, scores on the NCS-R decreased significantly after analgesia treatment, a finding that suggests that the NCS-R is a potentially valuable tool for pain management in patients with acquired brain injury. However, more studies are needed to determine the optimal approach to assessing pain in this population, given the individual variability in response to analgesia [39].

With regard to the NCS-R-I, we still need to determine the optimal time (before, during, and/or after the procedures) to administer the scale to identify differences in pain behaviors and in order to appropriately measure the pain associated with a given procedure. As other authors have noted, it is important to evaluate pain behavior after an intervention—whether pharmacological or non-pharmacological—in order to determine analgesic efficacy [11].

### 4.1. Limitations

We were unable to optimally assess convergent validity due to the lack of similar scales to assess correlation with NCS-R-I scores. Existing behavioral scales used to evaluate pain in critically ill patients have not been validated for use in patients with acquired brain injury. Some experts have suggested that these scales could be modified for use in patients with acquired brain injury, or a new scale based on those instruments could be developed [19,20,21]. Consequently, using these non-validated tools as comparators could compromise the validity of our findings, which is why we did not use them.

### 4.2. Relevance to Clinical Practice

Replication of psychometric studies in different contexts allows us to consolidate knowledge about pain assessment and management in patients with acquired brain injury. The availability of the NCS-R-I to detect and monitor pain behaviors associated with routine ICU procedures will allow clinicians to reliably assess pain and implement preventive strategies in this highly vulnerable patient population. Incorporating the NCS-R-I into standard ICU protocols could help standardize pain assessment and guide more effective analgesic interventions.

## 5. Conclusions

This study demonstrates that the psychometric properties of the Spanish version of the NCS-R-I make it suitable for use in patients who are unable to self-report pain due to brain injury and the presence of an artificial airway. The findings reported here confirm the clinical utility of this scale to assess and monitor nociceptive response in this patient population.

## Figures and Tables

**Table 1:** Semantic equivalence of the English and Spanish domains that were psychometrically validated.

**English version: Nociception Coma Scale-Revised-adapted for Intubated patients (NCS-R-I)**				
	0	1	2	3
FACIAL EXPRESSION	None	Oral reflexive movement/startle response	Grimace	Crying
MOTOR RESPONSE	None	Abnormal posturing	Flexion withdrawal	Localization to noxious stimulation
COMPLIANCE WITH VENTILATON	Tolerating ventilation	Coughing but tolerating ventilation most of the time	Fighting ventilator but ventilation possible sometimes	Unable to control ventilation
**Spanish version: Nociception Coma Scale-Revised-adapted for Intubated patients** **(NCS-R-I)**				
	0	1	2	3
RESPUESTAS DE EXPRESIÓN FACIAL	Ninguna	Movimiento reflejo oral/sobresalto	Muecas	Llanto
RESPUESTA MOTORA	Ninguna/flacidez	Postura anormal	Retirada con flexión	Localización a la estimulación dolorosa
ADAPTACIÓN A LA VENTILACIÓN	Tolera la ventilación	Tose pero tolera la ventilación la mayor parte del tiempo	Lucha con el ventilador pero a veces es posible la ventilación	Imposibilidad de controlar la ventilación

**Table 2:** Characteristics of the sample (n = 207).

Characteristics	Values
Age, years, median (IQR)	60 (46–68)
Sex (male), n (%)	138 (66.7)
Diagnosis, n (%) Cerebrovascular disease Traumatic brain injury Encephalopathy Other	110 (53.2) 76 (36.7) 10 (4.8) 11 (5.3)
Simplified Acute Physiology Score II, mean (SD)	49.6 (15.7)
Glasgow Coma Score, median (IQR)	9 (7–9)
Brain damage, n (%) Focal Diffuse	176 (85) 31 (15)
Neurosurgery, n (%)	81 (39.1)
Focal neurologic signs, n (%)	106 (52)
Days with mechanical ventilation, median (IQR)	14 (7–24)
Type of artificial airway, n (%) Endotracheal tube Tracheostomy cannula	171 (82.6) 36 (17.4)
Days between ICU admission and enrolment, median (IQR)	7 (3–13)
Days in ICU, median (IQR)	21 (12–33)
ICU mortality, n (%)	23 (11.1)
RASS, median (IQR)	−2 (−3–−2)
Continuous intravenous infusion ^a^, n (%) Fentanyl Morphine Remifentanil Propofol Midazolam Dexmedetomidine	61 (29.5) 45 (21.7) 29 (14) 72 (34.7) 8 (3.8) 15 (7.2)
Intravenous push infusion ^b^, mean (SD) Fentanyl Paracetamol Metamizole Dexketoprofen	19 (9.1) 76 (36.7) 37 (17.8) 13 (2.2)
Preemptive analgesic intervention prior to the start of procedures, n (%) <1 h 1–8 h	27 (13) 115 (55.5)

Abbreviations: SD, standard deviation; IQR, interquartile range; ICU: Intensive Care Unit; RASS: Richmond Agitation Sedation Scale. ^a^ Continuous intravenous infusion administered 24 h before pain assessment. ^b^ Intravenous push infusion administered up to 8 h prior to pain assessment.

**Table 3:** NCS-R-I: Ordinal alpha, interobserver agreement, and intraclass correlation coefficient.

Procedure		Ordinal Alpha		Interobserver Agreement (Kappa)			ICC	95% CI
		Observer 1	Observer 2	Domain 1	Domain 2	Domain 3		
Tracheal suction	Before	0.908	0.906	0.948	0.885	0.975	0.994	[0.992; 0.996]
	During	0.661	0.648	0.848	0.956	0.967	0.988	[0.984; 0.991]
	After	0.762	0.755	0.912	0.967	0.956	0.988	[0.985; 0.991]
Pressure-right nail bed	Before	0.772	0.772	1	1	1	0.997	[0.996; 0.998]
	During	0.708	0.727	0.925	0.961	0.983	0.993	[0.991; 0.995]
	After	0.833	0.833	0.939	1	0.972	0.991	[0.988; 0.993]
Pressure-left nail bed	Before	0.864	0.861	0.964	1	1	0.995	[0.994; 0.996]
	During	0.739	0.740	0.926	0.975	0.975	0.993	[0.991; 0.995]
	After	0.767	0.810	1	0.855	0.958	0.987	[0.983; 0.990]
Non-painful	Before	0.837	0.862	1	1	0.964	0.998	[0.997; 0.998]
	During	0.807	0.799	0.962	0.948	0.961	0.996	[0.995; 0.997]
	After	0.879	0.874	1	0.855	0.939	0.985	[0.980; 0.989]

Abbreviations: ICC: Intraclass Correlation Coefficient; CI: Confidence Interval.

**Table 4:** NCS-R-I score in different procedures, times, and observers.

			Observer 1		Observer 1	
			Mean (SD)	Median (IQR)	Mean (SD)	Median (IQR)
Tracheal suction	Before	Domain 1	0.18 (0.603)	0 (0–0)	0.19 (0.616)	0 (0–0)
		Domain 2	0.06 (0.290)	0 (0–0)	0.06 (0.290)	0 (0–0)
		Domain 3	0.18 (0.550)	0 (0–0)	0.18 (0.553)	0 (0–0)
		Total	0.42 (1.196)	0 (0–0)	0.43 (1.213)	0 (0–0)
	During	Domain 1	1.76 (1.140)	2 (1–3)	1.77 (1.124)	2 (1–3)
		Domain 2	1.39 (0.792)	1 (1–2)	1.41 (0.813)	1 (1–2)
		Domain 3	1.73 (0.719)	2 (2–2)	1.75 (0.727)	2 (2–2)
		Total	4.88 (1.982)	5 (3–6)	4.93 (1.974)	5 (4–6)
	After	Domain 1	0.19 (0.538)	0 (0–0)	0.18 (0.553)	0 (0–0)
		Domain 2	0.09 (0.299)	0 (0–0)	0.08 (0.292)	0 (0–0)
		Domain 3	0.23 (0.617)	0 (0–0)	0.21 (0.594)	0 (0–0)
		Total	0.50(1.070)	0 (0–0)	0.48 (1.065)	0 (0–0)
Pressure: right nail bed	Before	Domain 1	0.08 (0.353)	0 (0–0)	0.8 (0.353)	0 (0–0)
		Domain 2	0.01 (0.120)	0 (0–0)	0.01 (0.120)	0 (0–0)
		Domain 3	0.10 (0.423)	0 (0–0)	0.10 (0.423)	0 (0–0)
		Total	0.20(0.637)	0 (0–0)	0.21 (0.639)	0 (0–0)
	During	Domain 1	1.29 (1.120)	1 (0–2)	1.30 (1.127)	2 (0–2)
		Domain 2	0.31 (0.616)	0 (0–0)	0.30 (0.613)	0 (0–0)
		Domain 3	1.10 (0.968)	2 (0–2)	1.10 (0.973)	2 (0–2)
		Total	2.70 (2.036)	3 (1–4)	2.70 (2.069)	3 (1–4)
	After	Domain 1	0.11 (0.397)	0 (0–0)	0.13 (0.445)	0 (0–0)
		Domain 2	0.03 (0.195)	0 (0–0)	0.03 (0.195)	0 (0–0)
		Domain 3	0.17 (0.536)	0 (0–0)	0.16 (0.533)	0 (0–0)
		Total	0.31 (0.860)	0 (0–0)	0.32 (0.900)	0 (0–0)
Pressure: left nail bed	Before	Domain 1	0.12 (0.483)	0 (0–0)	0.13 (0.486)	0 (0–0)
		Domain 2	0 (0.70)	0 (0–0)	0 (0.70)	0 (0–0)
		Domain 3	0.09 (0.402)	0 (0–0)	0.9 (0.402)	0 (0–0)
		Total	0.22 (0.703)	0 (0–0)	0.23 (0.707)	0 (0–0)
	During	Domain 1	1.29 (1.155)	1 (0–2)	1.29 (1.155)	1 (0–2)
		Domain 2	0.35 (0.687)	0 (0–0)	0.35 (0.687)	0 (0–0)
		Domain 3	1.14 (0.954)	2 (0–2)	1.14 (0.949)	2 (0–2)
		Total	2.79 (2.146)	3 (1–5)	2.79 (2.148)	3 (1–5)
	After	Domain 1	0.09 (0.377)	0 (0–0)	0.09 (0.377)	0 (0–0)
		Domain 2	0.02 (0.138)	0 (0–0)	0.01 (0.120)	0 (0–0)
		Domain 3	0.10 (0.411)	0 (0–0)	0.09 (0.390)	0 (0–0)
		Total	0.21 (0.685)	0 (0–0)	0.20 (0.671)	0 (0–0)
Non-painful	Before	Domain 1	0.08 (0.374)	0 (0–0)	0.08 (0.374)	0 (0–0)
		Domain 2	0.03 (0.229)	0 (0–0)	0.03 (0.229)	0 (0–0)
		Domain 3	0.11 (0.416)	0 (0–0)	0.11 (0.421)	0 (0–0)
		Total	0.22 (0.718)	0 (0–0)	0.023 (0.783)	0 (0–0)
	During	Domain 1	0.25 (0.672)	0 (0–0)	0.26 (0.696)	0 (0–0)
		Domain 2	0.06 (0.297)	0 (0–0)	0.07 (0.320)	0 (0–0)
		Domain 3	0.23 (0.603)	0 (0–0)	0.22 (0.582)	0 (0–0)
		Total	0.55 (1.213)	0 (0–0)	0.55 (1.213)	0 (0–0)
	After	Domain 1	0.10 (0.441)	0 (0–0)	0.10 (0.441)	0 (0–0)
		Domain 2	0.01 (0.120)	0 (0–0)	0.02 (0.138)	0 (0–0)
		Domain 3	0.06 (0.328)	0 (0–0)	0.07 (0.335)	0 (0–0)
		Total	0.17 (0.630)	0 (0–0)	0.19 (0.645)	0 (0–0)

Abbreviations: SD, standard deviation; IQR, interquartile range.

**Table 5:** NCS-R-I score during the performance of nociceptive procedures compared to the scores observed before and after the performance of these procedures.

		NCS-R-I Score, Median (IQR)	*p* Value ^a^
Tracheal suction	Before vs. During	0 (0) vs. 5 (2.5)	<0.001
	During vs. After	5 (2.5) vs. 0 (0)	<0.001
	Before vs. After	0 (0) vs. 0 (0)	0.233
Pressure: right nail bed	Before vs. During	0 (0) vs. 3 (3)	<0.001
	During vs. After	3 (3) vs. 0 (0)	<0.001
	Before vs. After	0 (0) vs. 0 (0)	0.023
Pressure: left nail bed	Before vs. During	0 (0) vs. 3 (4)	<0.001
	During vs. After	3 (4) vs. 0 (0)	<0.001
	Before vs. After	0 (0) vs. 0 (0)	0.707

Abbreviations: IQR, interquartile range. ^a^ A correction for multiplicity has been considered, and therefore, a *p*-value < 0.017 will be considered significant.

## Data Availability

The datasets used and/or analysed during the current study are available from the corresponding author upon reasonable request.

## Public Involvement Statement

No public involvement in any aspect of this research.

## Guidelines and Standards Statement

This manuscript was drafted against the COnsensus-based Standards for the selection of health Measurement INstruments (COSMIN) [22] for reliability and validation research.

## Abbreviations

The following abbreviations are used in this manuscript:

ICU	Intensive Care Unit
NCS	Nociception Coma Scale
NCS-R	Nociception Coma Scale-Revised
NCS-R-I	Nociception Coma Scale-Revised-adapted for Intubated patients
COSMIN	COnsensus-based Standards for the selection of health Measurement INstruments
RASS	Richmond Agitation Sedation Scale
GCS	Glasgow Coma Scale
AUC	Area Under Curve
ROC	Receiver Operating Characteristic
ICC	Intraclass Correlation Coefficient
CI	Confidence Interval
